# Enhancement of Diosgenin Production in *Dioscorea zingiberensis* Cell Cultures by Oligosaccharides from Its Endophytic Fungus *Fusarium oxysporum *Dzf17 

**DOI:** 10.3390/molecules161210631

**Published:** 2011-12-19

**Authors:** Peiqin Li, Ziling Mao, Jingfeng Lou, Yan Li, Yan Mou, Shiqiong Lu, Youliang Peng, Ligang Zhou

**Affiliations:** Department of Plant Pathology, College of Agronomy and Biotechnology, China Agricultural University, Beijing 100193, China

**Keywords:** *Dioscorea zingiberensis*, *Fusarium oxysporium* Dzf17, diosgenin, oligosaccharides, polysaccharides, cell culture, elicitation

## Abstract

The effects of the oligosaccharides from the endophytic fungus *Fusarium oxysporum* Dzf17 as elicitors on diosgenin production in cell suspension cultures of its host *Dioscorea zingiberensis* were investigated. Three oligosaccharides, DP4, DP7 and DP10, were purified from the oligosaccharide fractions DP2-5, DP5-8 and DP8-12, respectively, which were prepared from the water-extracted mycelial polysaccharide of the endophytic fungus *F. oxysporum* Dzf17. When the cell cultures were treated with fraction DP5-8 at 20 mg/L on day 26 and harvested on day 32, the maximum diosgenin yield (2.187 mg/L) was achieved, which was 5.65-fold of control (0.387 mg/L). When oligosaccharides DP4, DP7 and DP10 were individually added to 26-day-old *D. zingiberensis* cell cultures at concentrations of 2, 4, 6, 8 and 10 mg/L in medium, DP7 at 6 mg/L was found to significantly enhance diosgenin production, with a yield of 3.202 mg/L, which was 8.27-fold of control. When the cell cultures were treated with DP7 twice on days 24 and 26, and harvested on day 30, both diosgenin content and yield were significantly increased and reached the maximums of 1.159 mg/g dw and 4.843 mg/L, both of which were higher than those of single elicitation, and were 9.19- and 12.38-fold of control, respectively.

## Abbreviations

DP: degree of polymerization
DP2-5: fraction composed of oligosaccharides with DPs from 2 to 5
DP5-8: fraction composed of oligosaccharides with DPs from 5 to 8
DP8-12: fraction composed of oligosaccharides with DPs from 8 to 12
DP4: oligosaccharide with DP as four
DP7: oligosaccharide with DP as seven
DP10: oligosaccharide with DP as ten; dw: dry weight
HPLC: high performance liquid chromatography
TFA: trifluoroacetic acid
TLC: thin-layer chromatography
WPS: water-extracted mycelial polysaccharide

## 1. Introduction

Diosgenin is a plant secondary metabolite, which has been extensively used in the pharmaceutical industry as a well-known precursor of various synthetic steroidal drugs [1]. Diosgenin has been reported to significantly increase both cholesterol content and cholesterol/phospholipid ratio in rat biliary lipids [2], to prevent cardiovascular disease [3], and to inhibit migration and invasion of human prostate cancer PC-3 cells by reducing expression of matrix metalloproteinases [4]. Diosgenin also showed an estrogenic effect on the mammary epithelium of ovariectomized (OVX) mouse [5], *in vitro* antiviral activity against hepatitis C virus [6], anti-proliferative and proapoptotic actions as a chemopreventive and therapeutic agent against several cancers [7], and anti-skin-aging effects [8]. Species from the genera *Balanites* (Zygophyllaceae) [9], *Costus* (Zingiberaceae) [10], *Dioscorea* (Dioscoreaceae) [1], and *Trigonella* (Leguminosae) [4] are the main plant sources for production of diosgenin, which is in the form of a steroidal saponin.

*Dioscorea zingiberensis *C. H. Wright is the dominant source for diosgenin production in China [11]. However, overexploitation of natural *D. zingiberensis* has led to a rapid decrease of this plant resource and a sharp shortage of diosgenin for pharmaceutical synthesis. Plant cell culture has been considered as an efficient and convenient alternative for the production of diosgenin [12]. The main barrier for commercialization of this technology is the low yield of diosgenin obtained in suspension cultures when plant cell culture systems are applied, therefore great efforts have been made seeking strategies for improvement of secondary metabolite production in plant cell cultures, such as selection of cell lines with high productivity, optimization of medium and culture conditions, application of genetic engineering and biotransformation, use of immobilization and permeabilization of cell cultures, and enhancement of secondary metabolite production by using elicitors [13]. Among these techniques, elicitation has attracted considerable attention in cell culture, because of its strong and rapid enhancement effects [14,15,16].

Elicitation is characterized by enhancement of secondary metabolite production with the elicitors which are classified as abiotic or biotic, depending on their origin [12,13]. Abiotic elicitors include ultraviolet irradiation, salts of heavy metals and other chemicals, while biotic elicitors refer to the substances obtained mainly from plants or microorganisms [13]. Nowadays, employment of fungal preparations as elicitors has become one of the most important and successful measures to enhance secondary metabolite production in plant cell cultures. Representative examples included *Silybum marianum* cell cultures for silymarin production [17], *Polygonum tinctorium *cell cultures for indirubin production [18], *Salvia miltiorrhiza* cell cultures for tanshinone production [19], and *Dioscorea galesttiana *cell cultures for diosgenin production [20]. Fungal elicitors were utilized mainly in the form of living or autoclaved mycelia, crude extracts, peptides, proteins and saccharides [12,13].

Both pathogenic and non-pathogenic fungi have been employed as the preparation sources of fungal elicitors. Fungal endophytes, being non-pathogenic fungi, have attracted more and more attention, because of protective effects towards pathogens and herbivores of their host plants. They are also potential sources of novel biologically active compounds [21,22,23]. However, there were few reports about the effects of endophytic fungi as elicitors on their host plants for secondary metabolite production [24]. *Fusarium oxysporum* Dzf17 is an endophytic fungus isolated from the rhizomes of *D. zingiberensis*. In our previous study the crude oligosaccharides prepared from the water-extracted mycelial polysaccharide of this fungus were preliminarily proven to show stimulating effects on diosgenin production in *D. zingiberensis* cell cultures [25]. It has also been demonstrated that the effects of oligosaccharides or polysaccharides on plant secondary metabolism depended on their composition, degree of polymerization (DP), time of addition, and concentration in medium [26]. The purpose of this work was to further fractionate the crude oligosaccharide prepared from the water-extracted mycelial polysaccharide of *F. oxysporum* Dzf17, and to investigate the effects of the purified oligosaccharides on cell growth and diosgenin production in *D. zingiberensis* suspension cell cultures.

## 2. Results

### 2.1. Effects of Oligosaccharide Fractions DP2-5, DP5-8 and DP8-12

**Figure 1:**
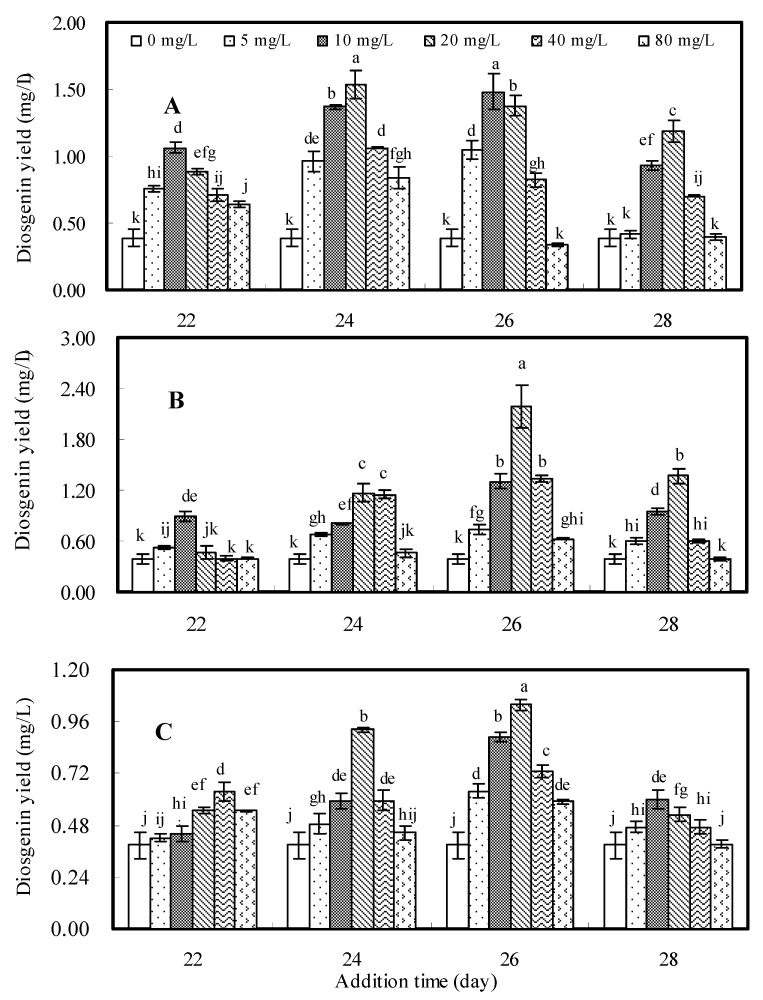
Effects of oligosaccharide fractions DP2-5 (**A**), DP5-8 (**B**) and DP8-12 (**C**) on diosgenin yield of *D. zingiberensis* cell cultures*.* The error bars represent standard deviations (*n* = 3). Different letters indicate significant differences among the treatments at *p* = 0.05 level.

Diosgenin yield (mg/L) in *D. zingiberensis* suspension cell cultures was the result of the synthesized diosgenin content (mg/g dw) and cell dry weight (g dw/L). Diosgenin content was found to contribute more to the diosgenin yield than cell dry weight in this study (data shown in the Supplementary Materials), so we may conclude that the variation of diosgenin yield was mainly diosgenin content-dependent. The effects of the oligosaccharide fractions DP2-5, DP5-8 and DP8-12 on diosgenin yield are presented in Figure 1. 

From the data the maximum diosgenin yield (2.187 mg/L) was obtained in the cell cultures treated with DP5-8 at 20 mg/L on day 26, which was 5.65-fold of control (0.387 mg/L). Similarly, the highest diosgenin yield (1.534 mg/L) for the cell cultures treated with DP2-5 at 20 mg/L on day 24 was found to be 3.96-fold of control, and the highest diosgenin yield (1.038 mg/L) for the cell cultures treated with DP8-12 at 20 mg/L on day 26 was found to be 2.68-fold of control. Among the three oligosaccharide fractions, DP5-8 was the most effective elicitor to stimulate diosgenin accumulation in *D. zingiberensis* cell cultures.

### 2.2. Effects of Oligosaccharides DP4, DP7 and DP10

**Figure 2:**
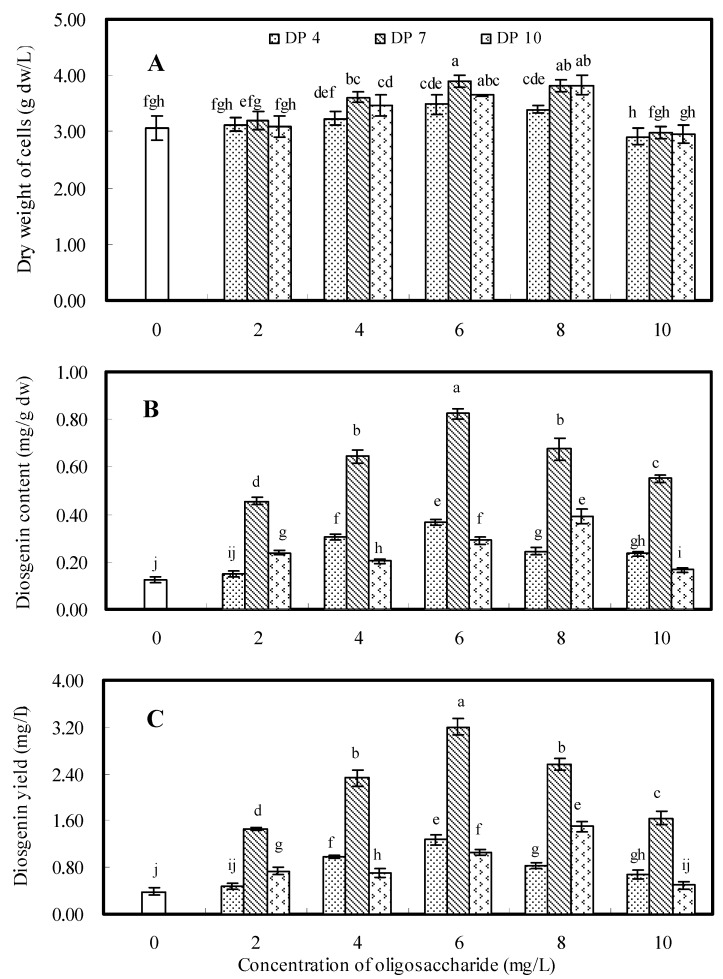
Effects of oligosaccharides DP4, DP7 and DP10 on dry weight (**A**), diosgenin content (**B**) and diosgenin yield (**C**) of *D. zingiberensis* cell cultures. The error bars represent standard deviations (*n* = 3). Different letters indicate significant differences among the treatments at *p* = 0.05 level.

Oligosaccharides DP4, DP7 and DP10 were purified from the oligosaccharide fractions DP2-5, DP5-8 and DP8-12, respectively. The effects of these three purified oligosaccharides on cell growth and diosgenin accumulation of *D. zingiberensis* cell cultures were further investigated with the results shown in Figure 2. The oligosaccharide elicitors were added on day 26, and the cell cultures were harvested on day 32. The effects of elicitors DP4, DP7 and DP10 on cell dry weight were shown in Figure 2A, from which no significant differences were observed between each treatment and the control when the cell cultures were treated with a lower dosage (*i.e*., 2 mg/L) of elicitors. However, cell growth was slightly inhibited at a higher dosage (*i.e*., 10 mg/L) of elicitors. When the cell cultures were individually treated with DP4, DP7 and DP10 at their corresponding concentrations of 6, 6 and 8 mg/L, the maximums of cell dry weight were 1.14-, 1.27- and 1.25-fold of control, respectively.

The effects of oligosaccharides DP4, DP7 and DP10 on diosgenin content of cell cultures are shown in Figure 2B, from which we can observe that diosgenin content was improved in all elicitation treatments. For the cell cultures treated with DP4 at 6 mg/L, the highest diosgenin content of 0.365 mg/g dw was observed, which was 2.90-fold of control (0.126 mg/g dw). For the cell cultures treated with DP10 at 8 mg/L, diosgenin content reached a maximum value (0.392 mg/g dw), which was 3.11-fold of control. Of all elicitation treatments, the highest diosgenin content (0.823 mg/g dw) was achieved by the addition of DP7 at 6 mg/L, which was 6.53-fold of control.

The effects of oligosaccharides DP4, DP7 and DP10 on diosgenin yield are shown in Figure 2C, which demonstrated that DP7 was the most effective elicitor to stimulate diosgenin accumulation. The maximum diosgenin yield (3.202 mg/L) was obtained in cell cultures treated with DP7 at 6 mg/L, which was 8.27-fold of control. When the cell cultures were treated with DP4 at 6 mg/L or DP10 at 8 mg/L, the maximum diosgenin yield observed was 1.273 mg/L and 1.498 mg/L, respectively. By comparing the effects caused by DP4, DP7 and DP10, it was concluded that oligosaccharide DP7 was the most effective elicitor to promote diosgenin accumulation in *D. zingiberensis* cell cultures.

### 2.3. Dynamics of Cell Growth and Diosgenin Accumulation Elicited once by DP7

**Figure 3:**
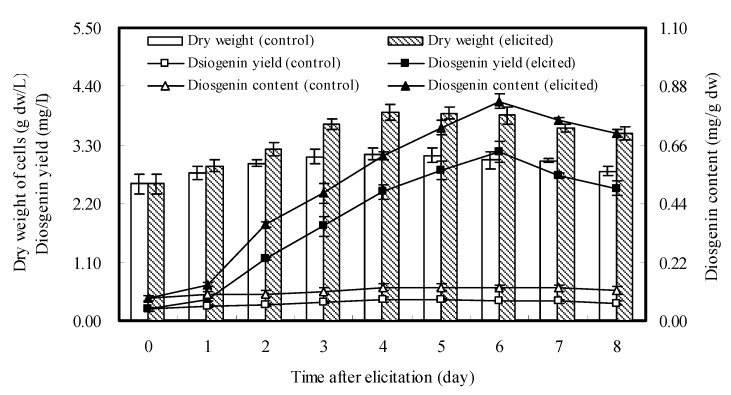
Time courses of growth and diosgenin accumulation of *D. zingiberensis *cell cultures treated once with DP7 added on day 26 at 6 mg/L. The error bars represent standard deviations (*n* = 3).

As shown in Figure 2, the maximum diosgenin production was obtained when *D. zingiberensis* cell cultures were treated with oligosaccharide DP7 at 6 mg/L on day 26 and harvested on day 32. To clarify the dynamics of cell growth and diosgenin accumulation of the cell cultures treated with DP7, the elicited cell cultures were harvested every day after addition of DP7, and both cell dry weight and diosgenin content were determined. The results (Figure 3) showed that dry weight of the elicited cell cultures was always higher than control during the days after elicitation, and reached the maximum (3.909 g dw/L) on day 30, and then remained almost constant until day 32. Diosgenin content of the elicited cell cultures began to increase significantly from the 2nd day after elicitation, and reached the maximum (0.823 mg/g dw) on day 32 and then decreased slightly. The varied trend of diosgenin yield was almost identical to that of diosgenin content, and the highest diosgenin yield (3.172 mg/L) was also observed on day 32. It was concluded that an optimal cultivation time for *D. zingiberensis* cell cultures to produce diosgenin by the addition of DP7 elicitor to the medium at 6 mg/L on day 26 after inoculation was set at 32 days.

### 2.4. Effects of Repeated Elicitation of DP7 on Cell Growth and Diosgenin Accumulation

Among three oligosaccharide elicitors, DP7 showed the best stimulating effect on diosgenin accumulation (shown in Figure 2). Hence, DP7 at concentration of 6 mg/L was chosen for further repeated elicitation, and the treated cell cultures were harvested on day 32. The effects of repeated elicitation on cell growth and diosgenin accumulation were summarized in Table 1. Cell growth was significantly improved as compared with the control when the cell cultures were treated once or twice with DP7 at 6 mg/L. The maximum diosgenin content (0.931 mg/g dw) was observed in the cell cultures elicited twice on days 24 and 26, which was higher than that (0.821 mg/g dw) of single elicitation. Correspondingly, the highest diosgenin yield (3.548 mg/L) was gained in the cell cultures treated twice with DP7 at 6 mg/L added on days 24 and 26, which was 9.16-fold of control and exhibited significant enhancement as compared with that of single elicitation. Thus, twice repeated elicitation with DP7 added on days 24 and 26 with addition dose of 6 mg/L could be carried out to effectively stimulate diosgenin accumulation.

**Table 1:** Effects of repeated elicitation with DP7 at 6 mg/L on growth and diosgenin accumulation of *D. zingiberensis* cell cultures.

Addition time	Dry weight	Diosgenin content	Diosgenin yield
(day)	(g dw/L)	(mg/g dw)	(g/L)
Control (without elicitation)	3.070 ± 0.217 c	0.126 ± 0.012 d	0.387 ± 0.061 d
Days 24 and 26	3.809 ± 0.164 a	0.931 ± 0.039 a	3.548 ± 0.280 a
Day 26	3.898 ± 0.143 a	0.821 ± 0.029 b	3.203 ± 0.222 b
Days 26 and 28	3.332 ± 0.057 b	0.733 ± 0.021 c	2.440 ± 0.030 c

Note: The values represent means ± standard deviations (*n* = 3). Different letters indicate significant differences among the treatments in each column at *p* = 0.05 level.

**Figure 4:**
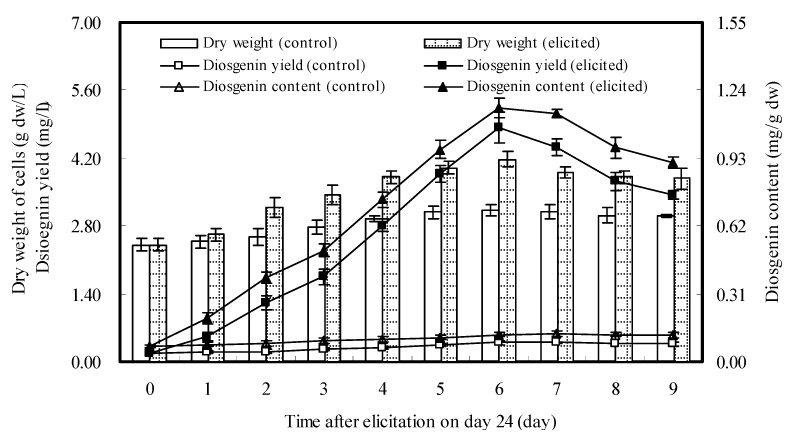
Time courses of growth and diosgenin accumulation of *D. zingiberensis* cell cultures treated twice with DP7 added on days 24 and 26 at 6 mg/L. The error bars represent standard deviations (*n* = 3).

The optimal harvest time for the cell cultures elicited once was determined on day 32. In order to determine the appropriate harvest time for the cell cultures by repeated elicitation, time courses of growth and diosgenin accumulation of *D. zingiberensis* cell cultures elicited twice with DP7 added on days 24 and 26 were further investigated, and the results were shown in Figure 4. 

When the cell cultures were treated twice with DP7 and harvested on day 30, the cell dry weight reached a maximum value (4.177 g dw/L), which was greater than that (3.809 g dw/L) of the cell cultures harvested on day 32. Diosgenin content of the cell cultures elicited twice increased almost linearly from day 24 up to day 30, and reached a maximum value (1.159 mg/g dw) on day 30, which was higher than that (0.931 mg/g dw) of the cell cultures harvested on day 32, and was 9.19-fold of control (without elicitation). Consequently, the maximum diosgenin yield (4.843 mg/L which was 12.38-fold of control) of the cell cultures elicited twice was observed on day 30. Thus, repeated elicitation with DP7 added on days 24 and 26 with an addition dosage of 6 mg/L and harvested on day 30 could lead to considerable accumulation of diosgenin in *D. zingiberensis *cell cultures, and showed stronger enhancement than that of single elicitation.

## 3. Discussion

It has been conclusively demonstrated that application of fungal elicitors could effectively promote secondary metabolite accumulation in plant cell culture system [12,13]. Endophytic fungi have been considered as a new group of fungi and received much attention from investigators very recently [27]. They have been proved to protect their host plants from the attacks of pathogens and herbivores, and have been regarded as the sources of natural products of pharmaceutical and agricultural importance [28]. In the present study, the endophytic fungus *F. oxysporum* Dzf17 was employed to prepare oligosaccharide elicitors that when applied in suspension cell cultures of its host plant *D. zingiberensis*, could not only realize effective enhancement of diosgenin accumulation in *D. zingiberensis* cell cultures but also provide a basis for further investigation of the interactions between each fungal endophyte and its host plant.

Effects of oligosaccharide elicitors on plant cell growth and accumulation of secondary metabolites have been considered to be related to their DPs [29]. Among three oligosaccharide fractions in this study, DP5-8 was found as the most effective elicitor to stimulate diosgenin accumulation in *D. zingiberensis* cell cultures. Diosgenin yield of the cells treated with the elicitor DP5-8 under the optimal elicitation condition was 5.65-fold of control, which was far higher than that of the crude oligosaccharide (3.26-fold) [25]. Three oligosaccharides DP4, DP7 and DP10 were individually prepared from oligosaccharide fractions DP2-5, DP5-8 and DP8-12. The optimal enhancement of diosgenin production caused by DP4 was weaker than that of DP2-5, which suggested that DP4 could not be the main effective component in oligosaccharide fraction DP2-5. Either DP7 or DP10 showed stronger enhancement of diosgenin accumulation than that of DP5-8 or DP8-12, respectively, which indicated that DP7 or DP10 was the main effective component in its corresponding oligosaccharide fraction. Based on the results in this study, it was concluded that an elicitation effect could be determined by the elicitor's properties along with its addition time and concentration.

The limited duration of an elicitor’s effect on secondary metabolite accumulation has been observed in plant cell cultures, and it might be related to the limitation stimulation of the activities of key enzymes for biosynthesis of secondary metabolites [30,31,32]. This case was also found in present study with a single elicitation of DP7. When *D. zingiberensis *cell cultures were treated twice with DP7 added at 6 mg/L on days 24 and 26, diosgenin yield was increased far more than by a single elicitation. Time courses of cell growth and diosgenin accumulation in *D. zingiberensis* cell cultures treated once or twice with DP7 were individually investigated (Figure 3 and Figure 4), from which we observed that diosgenin accumulation in the cell cultures elicited twice reached a maximum value (4.843 mg/L) on day 30, two days ahead as compared with that of single elicitation. Hence, it suggested that the optimal harvested time for the cell cultures treated once or twice may vary with the number of elicitations. In this study, diosgenin accumulation was significantly enhanced by the oligosaccharide elicitors from water-extracted mycelial polysaccharide of endophytic *F. oxysporum* Dzf17, especially when elicitation experiment was conducted once or twice with oligosaccharide DP7 at 6 mg/L, which showed satisfactory enhancement effects. The observations from this study were in good agreement with those reported previously [30,31,32]. In this study, we only determined the content and yield of diosgenin in *D. zingiberensis* cell cultures. As diosgenin is the sapogenin of the steroidal saponins in *D. zingiberensis*, it is necessary to understand which saponin biosynthesis in the cultures is stimulated by DP7.

## 4. Experimental

### 4.1. Cell Suspension Cultures

The calli of *D. zingiberensis *C. H. Wright were induced and established as described previously [25]. They were subsequently subcultured on Murashige and Skoog (MS) medium [33] supplemented with 6-benzyladenine (1.5 mg/L), naphthalene acetic acid (1.0 mg/L), agar (8 g/L), and sucrose (30 g/L) at an interval of 30 days. The medium pH was adjusted to 5.8 before autoclaving for 15 min at 121 °C. The cell suspension cultures, which were established and maintained on the above liquid medium at a subculture interval of 4 weeks, were used as inoculums for the experimental flasks. All experiments were carried out in 125-mL Erlenmeyer flasks, each of which contained 30 mL of liquid medium inoculated with 0.3 g fresh weight of 21-day-old cell suspension cultures. The Erlenmeyer flasks were incubated in darkness on a rotary shaker at 120 rpm and 25 °C.

### 4.2. Preparation of Oligosaccharides

The water-extracted mycelial polysaccharide of *F. oxysporum* Dzf17 was prepared by ethanol precipitation as described previously [34]. The water-extracted mycelial polysaccharide (20 g) was subjected to hydrolysis with 2.17 mol/L TFA (250 mL) at 85 °C for 4 h, and the acid hydrolytes were then filtrated. TFA in the filtrate was evaporated in the form of an azeotrope with methanol by vacuum concentration. The filtrate was concentrated and designated as the crude oligosaccharide.

The crude oligosaccharide (4.785 g) was fractionated by Bio-Gel P2 column chromatography and eluted with distilled water. Each 10 mL of eluate was collected in a small bottle, lyophilized, and then subjected to thin-layer chromatography (TLC) detection with *n*-butanol-ethyl acetate-acetic acid-water (3.0:0.5:1.7:4.1, v/v) as the developing agent. Three main fractions were obtained as compared with the reference oligosaccharides, which were kindly supplied by Prof. Shilin Wang of Kunming Institute of Botany, Chinese Academy of Sciences. Their degrees of polymerization (DPs) were at ranges of 2 to 5, 5 to 8, and 8 to 12, respectively. Therefore, they were designated as the fractions DP2-5 (724.1 mg), DP5-8 (748.7 mg) and DP8-12 (864.3 mg). Each fraction (*i.e*., DP2-5, DP5-8 and DP8-12, 500 mg) was further fractionated by Bio-Gel P2 chromatography, and three purified oligosaccharides were obtained, respectively. By TLC detection and electro-spray ionization-mass spectrometry (ESI-MS) analysis, the DP of each purified oligosaccharide was determined (data shown in the supplementary materials). Three oligosaccharides were named as oligosaccharides DP4 (15.3 mg), DP7 (26.7 mg) and DP10 (18.3 mg).

### 4.3. Elicitation Treatment of the Suspension Cells

Both the above oligosaccharide fractions (*i.e*., DP2-5, DP5-8 and DP8-12) and purified oligosaccharides (*i.e.*, DP4, DP7 and DP10) were separately dissolved in distilled water as the concentrated stock solutions, then filter-sterilized through a 0.45 μm membrane and stored at 4 °C until required. Oligosaccharide fractions DP2-5, DP5-8 and DP8-12 at concentrations of 5, 10, 20, 40 and 80 mg/L in medium were individually added to 22-, 24-, 26- and 28-day-old *D. zingiberensis *suspension cell cultures. The suspension cell cultures were harvested on day 32, and then both dry weight and diosgenin content of cell cultures were determined. The optimal oligosaccharide fraction along with its addition time and concentration was screened.

The three purified oligosaccharides DP4, DP7 and DP10 were further investigated for their effects on cell growth and diosgenin accumulation in *D. zingiberensis* cell cultures. They were individually added to 26-day-old cell cultures at concentrations of 2, 4, 6, 8 and 10 mg/L. Furthermore, repeated elicitations by oligosaccharide DP7 at 6 mg/L were developed. DP7 was repeatedly added to the cell cultures either on days 24 and 26 or on days 26 and 28. Time courses of the cell growth and diosgenin accumulation were investigated when the cell cultures were treated with DP7 at 6 mg/L once or twice.

### 4.4. Determination of Cell Dry Weight

The suspension cell cultures were harvested, and separated from the liquid medium by filtration, washed with distilled water to remove residual medium, and then filtrated again under vacuum to obtain fresh cells, which were further lyophilized to a constant dry weight (dw) and expressed as gram per liter.

### 4.5. Diosgenin Extraction and Quantification

Diosgenin extraction was carried out as described previously with some modifications [35]. Briefly, powdered dry cultured cells (100 mg) was added into a tube with 95% ethanol (20 mL), and then subjected to ultrasonic extraction for 1 h. After that, 1 mol/L sulfuric acid (20 mL) was added to each tube, and hydrolyzed at 121 °C for 2 h. The hydrolyte was extracted for three times with petroleum ether. The combined petroleum ether solution was washed twice with 1 mol/L of NaOH solution, and then twice with distilled water. After dehydration with anhydrous sodium sulfate, the petroleum ether solution was then concentrated to dryness under vacuum on a rotary evaporator. The extract was dissolved in acetonitrile, and then filtered through a 0.22 μm filter before analysis.

Diosgenin quantification was carried out by a high performance liquid chromatography system (Shimadzu, Japan), which consisted of two LC-20AT high-pressure solvent delivery pump units, an SPD-M20A photodiode array detector (PAD), an SIL-20AC autosampler, CTO-10AS column oven, and CBM-20Alite system controller. A reversed-phase Agilent TC-C_18_ column (250 mm × 4.6 mm i.d., 5 μm particle size) was used for separation by using a mobile phase of acetonitrile-water (90:10, v/v) at a flow rate of 1 mL/min at 30 °C. LCsolution multi-PDA workstation was employed to acquire and process chromatographic data. The injection volume was 20 μL. Changes in absorbance at 203 nm were recorded. The peak area was calibrated to diosgenin content with a chemical standard (Sigma). Diosgenin content in the culture medium was negligible and not determined.

### 4.6. Statistical Analysis

All treatments were performed in triplicate, and the results were represented by their mean values and the standard deviations (SD). The data were analyzed to detect significant differences by PROC ANOVA of SAS version 8.2. The term significant has been used to denote the differences for which *p* ≤ 0.05.

## 5. Conclusions

In this work, oligosaccharide elicitors prepared from the endophytic fungus *F. oxysporum* Dzf17 were found to significantly enhance diosgenin accumulation in *D. zingiberensis* cell cultures. The stimulating effects were related to the properties, addition time and concentration of each oligosaccharide along with the harvesting time of the treated cell cultures. Among the oligosaccharide fractions, DP5-8 was the most effective elicitor to stimulate diosgenin accumulation in *D. zingiberensis *cell cultures treated on day 26 at 20 mg/L and harvested on day 30, diosgenin yield was 5.65-fold of control. Furthermore, the effects of three purified oligosaccharides DP4, DP7 and DP10, respectively isolated from DP2-5, DP5-8 and DP8-12, on diosgenin accumulation were also investigated. The highest diosgenin yield in the cell cultures was enhanced by DP7 at 6 mg/L added on day 26, which was 8.27-fold of control. However, repeated elicitations with DP7 at 6 mg/L added on days 24 and 26 appeared more desirable effects on diosgenin accumulation. Meanwhile, diosgenin yield in twice elicited cell cultures was significantly increased to 12.38-fold of control. However, the mechanisms whereby the oligosaccharides regulate plant growth or elicit biosynthesis of plant secondary metabolites are poorly understood, and are extremely worthy of further study [26]. As a whole, the application of oligosaccharide elicitors from endophytic fungus *F. oxysporum* Dzf17 to stimulate diosgenin accumulation in *D. zingiberensis* cell cultures could be an effective strategy for large-scale production of diosgenin in the future. Furthermore, it is beneficial for us to further understand the interactions between each endophytic fungus and its host plant, and fully exploit and utilize endophytic fungal resources. The cost for the production of diosgenin by using oligosaccharide DP7 should be another aspect to be considered in the future.

## Supplementary Materials

Supplementary materials can be accessed at: http://www.mdpi.com/1420-3049/16/12/10631/s1.
