# Supplementary material for: Enhancement of Diosgenin Production in Dioscorea zingiberensis Cell Cultures by Oligosaccharides from Its Endophytic Fungus Fusarium oxysporum Dzf17
Source: Molecules. 2011 Dec 19;16(12):10631–44. doi: 10.3390/molecules161210631 (PMC6264283; doi:10.3390/molecules161210631)
Supplement: Supplementary file 1 [file molecules-16-10631-s001.doc]

Enhancement of Diosgenin Production in *Dioscorea zingiberensis* Cell Cultures by Oligosaccharides from its Endophytic Fungus *Fusarium oxysporum* Dzf17

Peiqin Li, Ziling Mao, Jingfeng Lou, Yan Li, Yan Mou, Shiqiong Lu, Youliang Peng and
Ligang Zhou *

Department of Plant Pathology, College of Agronomy and Biotechnology, China Agricultural University, Beijing 100193, China

* Author to whom correspondence should be addressed; E-Mail: lgzhou@cau.edu.cn;
Tel.: +86-10-62731199.

Supplementary Materials

S1. Deduction of Degree of Polymerization of Purified Oligosaccharides DP4, DP7 and DP10

Three oligosaccharides were repeatedly purified by Bio-Gel P2 chromatography from three oligosaccharide fractions DP2-5, DP5-8 and DP8-12, respectively. Each oligosaccharide displayed a single spot by TLC detection. By comparing their R_f_ values with the standard oligosaccharide references, their degrees of polymerization (DPs) could be preliminarily determined as 4, 7 and 10, so they were named as DP4, DP7 and DP10, respectively. Further deduction of DP was carried out by ESI-MS. Their positive ion (Na^+^) peaks by ESI-MS are presented in Figure S1.

**Figure S1.** Positive ion (Na^+^) ESI-MS spectra of oligosaccharides DP4 (**A**), DP7 (**B**) and DP10 (**C**).

**A**

**Figure S1.** *Cont*.

**B**

**C**

Fragment ion peaks with fair intensity in the MS spectrum of DP4 originating from a pseudo molecular ion [M+Na]^+^ peak at *m/z* 725.3 were observed, corresponding to the sequential loss of one monosaccharide unit (*m/z* 545.2) and two monosaccharide units (*m/z* 383.1), which are presented in Figure S1A. The fragment ion at *m/z* 383.1 could be separated into two monosaccharide units and one Na^+^. Thus we deduced oligosaccharide DP4 was composed of four monosaccharide units. Its degree of polymerization was 4, hence it was designated as DP4. The strong pseudo molecular ion [M+Na]^+^ peaks of DP7 and DP10 were shown at *m/z* 1175.5 and *m/z* 1662.8, which were respectively presented in Figures S1B and S1C. Their molecular weights were presumed to be 1152.5 and 1639.6, which were approximately the correct molecular weights for a heptasaccharide (1152) and a decasaccharide (1639), respectively. Therefore, the DPs of these two oligosaccharides were deduced as 7 and 10, and they were then designated as DP7 and DP10, respectively.

Completely hydrolysis of each oligosaccharide was conducted by using 2.17 mol/L of trifluoroacetic acid for 2 h at 121 °C. TLC detections of hydrolysates of all three purified oligosaccharides were generally identical to that of standard glucose. So the oligosaccharides were all composed of glucose. It is dramatically difficult to determine the accurate structures (*i.e*., homogeneity, and linkage of the monosaccharide residues) of the oligosaccharides. Therefore, this research was mainly focused on their physiological effects on plant suspension cell cultures. The effects of the oligosaccharides with various DPs on cell growth and diosgenin accumulation in *Dioscorea zingiberensis* cell cultures were compared in detail.

S2. Effects of Oligosaccharide Fractions DP2-5, DP5-8 and DP8-12 on Cell Growth of Suspension Cultures of D. zingiberensis

Oligosaccharide fractions DP2-5, DP5-8 and DP8-12 were individually added to 22-, 24-, 26- and 28-day-old suspension cell cultures of *D. zingiberensis*, at concentrations of 5, 10, 20, 40, and 80 mg/L. The cell cultures were harvested on day 32. The effects of oligosaccharide fractions DP2-5, DP5-8 and DP8-12 on growth of cell cultures were respectively presented in Figure S2. When the cell cultures were treated with the elicitors either on day 22 or on day 28, no obvious enhancement of the cell growth was observed except that the treatment with DP5-8 at 20 mg/L added on day 28 showed slight promoting effect. However, cell dry weight was significantly increased in a dose-dependent manner when all the three elicitors were added on day 24 or on day 26. The optimal elicitation treatments for promoting cell growth were at 20 mg/L on day 24 for DP2-5, 20 mg/L on day 26 for DP5-8, and
20 mg/L on day 24 for DP8-12, respectively. Under the optimal elicitation conditions, dry weights of the cell cultures treated with DP2-5, DP5-8 and DP8-12 were 1.20-, 1.33- and 1.34-fold of control (3.070 g dw/L), respectively.

**Figure S2.** Effects of oligosaccharide fractions DP2-5 (A), DP5-8 (B) and DP8-12 (C) on dry weight of *D. zingiberensis* cell cultures. The error bars represent standard deviations (*n* = 3). Different letters indicate significant differences among the treatments at *p* = 0.05 level.

**A**

**B**

**C**

S3. Effects of Oligosaccharide Fractions DP2-5, DP5-8 and DP8-12 on Diosgenin Content in Suspension Cell Cultures of D. zingiberensis

The effects of oligosaccharide fractions DP2-5, DP5-8 and DP8-12 on diosgenin content were shown in Figure S3. No inhibitory effect on diosgenin content was observed when cell cultures were treated with the oligosaccharide fractions. When elicitor DP2-5 was added at 20 mg/L on day 24 and at 10 mg/L on day 26, the values of diosgenin content were 0.417 mg/g dw and 0.409 mg/g dw, which were 3.32- and 3.26-fold of control (0.126 mg/g dw), respectively. The maximum diosgenin content (0.537 mg/g dw) was observed in the cultured cells treated with DP5-8 at 20 mg/L on day 26, which was 4.27-fold of control.

**Figure S3.** Effects of oligosaccharide fractions DP2-5 (A), DP5-8 (B) and DP8-12 (C) on diosgenin content of *D. zingiberensis* cell cultures. The error bars represent standard deviations (*n* = 3). Different letters indicate significant differences among the treatments at *p* = 0.05 level.

**A**

**B**

**C**

Similarly, the maximum diosgenin content (0.280 mg/g dw) was observed in the cultured cells treated with DP8-12 at 20 mg/L on day 26, which was 2.23-fold of control. We concluded that the optimal addition time for three oligosaccharide fractions to increase diosgenin content was on day 24 or on day 26. Among them, DP5-8 added at 20 mg/L on day 26 was the most effective elicitor to increase diosgenin content in *D. zingiberensis* cell cultures.
